# Impairment of the *Plasmodium falciparum* Erythrocytic Cycle Induced by Angiotensin Peptides

**DOI:** 10.1371/journal.pone.0017174

**Published:** 2011-02-18

**Authors:** Victor Barbosa Saraiva, Leandro de Souza Silva, Claudio Teixeira Ferreira-DaSilva, João Luiz da Silva-Filho, André Teixeira-Ferreira, Jonas Perales, Mariana Conceição Souza, Maria das Graças Henriques, Celso Caruso-Neves, Ana Acacia de Sá Pinheiro

**Affiliations:** 1 Instituto Federal de Educação, Ciência e Tecnologia Fluminense, Cabo Frio, Brazil; 2 Instituto de Biofísica Carlos Chagas Filho, Universidade Federal do Rio de Janeiro, Rio de Janeiro, Brazil; 3 Instituto Oswaldo Cruz, Fundação Oswaldo Cruz, Rio de Janeiro, Brazil; 4 Rede Proteômica do Rio de Janeiro, Rio de Janeiro, Brazil; 5 Instituto de Tecnologia em Fármacos, Fundação Oswaldo Cruz, Rio de Janeiro, Brazil; 6 Instituto Nacional de Ciência e Tecnologia em Biologia e Bioimagem, Conselho Nacional de Desenvolvimento Científico e Tecnológico/MCT, Rio de Janeiro, Brazil; 7 Instituto Nacional para Pesquisa Translacional em Saúde e Ambiente na Região Amazônica, Conselho Nacional de Desenvolvimento Científico e Tecnológico/MCT, Rio de Janeiro, Brazil; Université Pierre et Marie Curie, France

## Abstract

*Plasmodium falciparum* causes the most serious complications of malaria and is a public health problem worldwide with over 2 million deaths each year. The erythrocyte invasion mechanisms by *Plasmodium* sp. have been well described, however the physiological aspects involving host components in this process are still poorly understood. Here, we provide evidence for the role of renin-angiotensin system (RAS) components in reducing erythrocyte invasion by *P. falciparum*. Angiotensin II (Ang II) reduced erythrocyte invasion in an enriched schizont culture of *P. falciparum* in a dose-dependent manner. Using mass spectroscopy, we showed that Ang II was metabolized by erythrocytes to Ang IV and Ang-(1–7). Parasite infection decreased Ang-(1–7) and completely abolished Ang IV formation. Similar to Ang II, Ang-(1–7) decreased the level of infection in an A779 (specific antagonist of Ang-(1–7) receptor, MAS)-sensitive manner. 10^−7^ M PD123319, an AT_2_ receptor antagonist, partially reversed the effects of Ang-(1–7) and Ang II. However, 10^−6^ M losartan, an antagonist of the AT_1_ receptor, had no effect. Gs protein is a crucial player in the *Plasmodium falciparum* blood cycle and angiotensin peptides can modulate protein kinase A (PKA) activity; 10^−8^ M Ang II or 10^−8^ M Ang-(1–7) inhibited this activity in erythrocytes by 60% and this effect was reversed by 10^−7^ M A779. 10^−6^ M dibutyryl-cAMP increased the level of infection and 10^−7^ M PKA inhibitor decreased the level of infection by 30%. These results indicate that the effect of Ang-(1–7) on *P. falciparum* blood stage involves a MAS-mediated PKA inhibition. Our results indicate a crucial role for Ang II conversion into Ang-(1–7) in controlling the erythrocytic cycle of the malaria parasite, adding new functions to peptides initially described to be involved in the regulation of vascular tonus.

## Introduction

Malaria, one of the most severe parasitic diseases, is caused by *Plasmodium* sp. More than 100 species that can infect vertebrates have been identified in nature and *Plasmodium falciparum* is the most lethal among them [1]. Although there have been many efforts to control the disease, it remains a serious public health problem worldwide, especially because of multidrug resistance mechanisms in parasites, development of insecticide resistance in mosquitoes and the absence of an effective vaccine [2]. This infection affects mainly the poor population, and morbidity and mortality can be attributed to the lack of adequate treatment and an early and accurate diagnosis, constituting an obstacle to efforts toward economic development [2].

The life cycle of the malaria parasite is extremely complex and is characterized by an asexual phase, which occurs inside the vertebrate host, and a sexual phase that develops in the mosquito vector [3]. The clinical symptoms of the disease usually coincide with disruption of infected erythrocytes, followed by the release of merozoites in the circulation, which can infect new red blood cells, perpetuating the parasite erythrocytic cycle in the asexual phase [3].

The mechanism of erythrocyte invasion by merozoites has been studied by several groups and it is characterized as a multi-step process involving host erythrocyte membrane involution and deformation, leading to invagination and cell swallowing [4], [5]. For many years, this mechanism of entry into erythrocytes was considered to be mediated by parasite factors specially secreted by apical organelles. Today, it is known that components enriched in lipid rafts from host erythrocyte membrane are also involved in this process. Heterotrimeric G protein, specifically the Gs α subunit (Gαs), is present on erythrocyte detergent-resistant membrane rafts and is recruited to the parasitophorous vacuole [6], [7]. Harrison et al. [8] showed the involvement of erythrocyte G-protein raft-associated signaling mechanisms in parasite entry. Agonists of adenosine and β-adrenergic receptors, two well-known G protein-coupled receptors that are also recruited to the plasmodial vacuole membrane, stimulated infection; antagonists reversed these agonist effects. Murphy et al. [9], by using erythrocyte ghosts, provided final evidence that erythrocyte Gs signaling is important not only to invasion but also to parasite intracellular maturation. They observed that the inhibition of Gs protein prevents the increase in the production of cAMP induced by isoproterenol and parasite invasion into erythrocytes [9]. Because cAMP is a well-known activator of protein kinase A (PKA) activity, it is possible to postulate the involvement of this kinase in the erythrocytic cycle of the malaria parasite. Furthermore, the presence of PKA has been described in *Plasmodium falciparum* (PfPKA), which is also involved in parasite invasion [10]. Thus, based on these observations we can postulate that peptides that can bind and signal through a member of the G protein-coupled receptor family (GPCR) could participate in erythrocyte invasion by *Plasmodium* sp.

A possible candidate is angiotensin II (Ang II), a peptide that belongs to the renin-angiotensin system (RAS), which has confirmed proinflammatory effects and antiplasmodial activity when injected in the hemolymph of *Aedes aegypti* contaminated with *Plasmodium gallinaceum* by blocking accumulation of sporozoites in mosquito salivary glands without affecting vector survival [11]. Furthermore, a correlation between mild malaria and patients with angiotensin I converting enzyme (ACE I/D) and angiotensin II converting enzyme (ACE2 C→T) has been observed [12]. The RAS has been shown to be present in different cells of the hematopoietic system and it seems that Ang II stimulates erythropoiesis by up-regulating erythropoietin levels and by direct stimulation of the proliferation of erythroid progenitor cells [13]. It seems that Ang II receptors have not been characterized in fully differentiated erythrocytes and direct evidence of their participation on *P. falciparum* entry is scarce. In this report, we describe studies aimed at identifying the molecular mechanisms induced by Ang II that are involved in the modulation of the *P. falciparum* erythrocytic cycle.

## Results

### Ang II impairs the P. falciparum erythrocytic cycle

**Figure 1:**
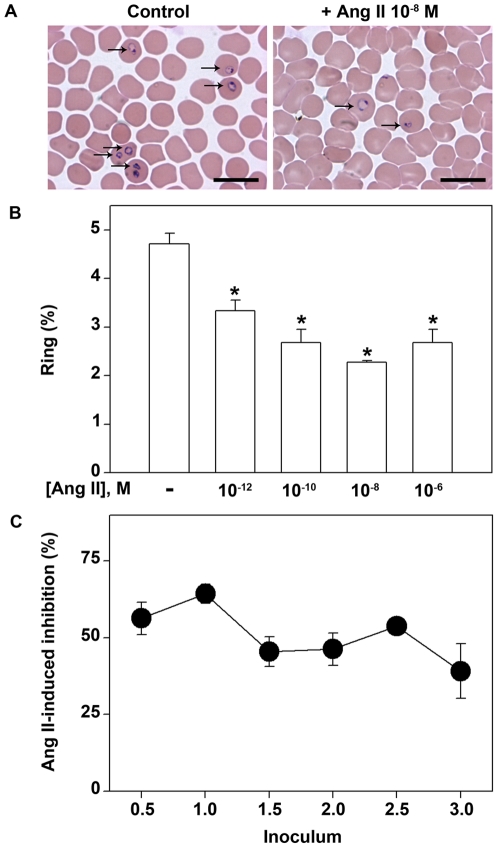
Angiotensin II reduces erythrocyte invasion by *Plasmodium falciparum*. (A) Representative thick blood smears for parasitemia determination by Diff-Quick staining. Parasite schizont forms were incubated with a fresh erythrocyte culture at 2% parasitemia in the absence (left) or presence (right) of 10^−8^ M Ang II (n = 8). (B) Parasite schizont forms were incubated with a fresh erythrocyte culture at 2% parasitemia in the absence or presence of increasing concentrations of Ang II (10^−12^–10^−6^ M). Parasite invasion was determined by the number of intracellular rings after 24 h incubation as described in the Materials and Methods section (n = 8). (C) The influence of Ang II was evaluated in different inoculum of parasite schizont forms (from 0.5% to 3%), 24 h after the initial inoculum, by determining parasite invasion in thick blood smears as described in the Materials and Methods section (n = 6). The results are expressed as means±SE. *Statistically significant compared with the control value (*P*<0.05). Magnification ×100.

To assess the effect of Ang II in the *P. falciparum* erythrocytic cycle, increasing concentrations of the peptide (10^−12^–10^−6^ M) were assayed in vitro against a synchronized culture in the schizont form of the W2 strain of *P. falciparum* maintaining 5% hematocrit and 2% parasitemia. Twenty-four hours after treatment, Ang II was able to reduce parasite invasion in a dose-dependent manner with the maximum effect observed at 10^−8^ M, promoting 47% of inhibition (Fig. 1A,B). The schizont and trophozoite forms represent less than 10% of total parasites after 24 h. Fig. 1C shows the correlation between the Ang II-induced inhibition of parasite invasion and initial inoculum of synchronized culture in the schizont form of the W2 strain, using increasing parasitemia from 0.5 to 3%. The inhibitory effect of 10^−8^ M Ang II was not changed by different inoculum.

**Figure 2:**
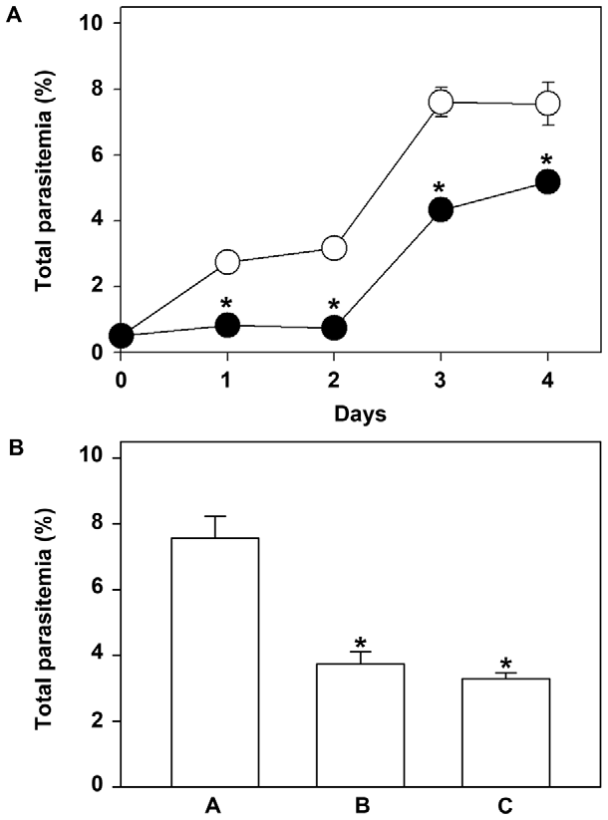
Ang II inhibitory effect is maintained for 11 days. (A) Parasites were incubated with a fresh erythrocyte culture at 0.5% parasitemia in the absence (○) or presence (•) of 10^−8^ M Ang II, added daily for 4 consecutive days. Total parasitemia was determined on each day of the assay as described in the Materials and Methods section (n = 7). (B) The pre-incubation effect of Ang II was determined after treating the culture without (bar A) or with (bar B) 10^−8^ M Ang II for 4 days. The culture was then split and maintained for 11 days without Ang II (bar C). The total parasitemia was determined as described in the Materials and Methods section (n = 5). The results are expressed as means±SE. *Statistically significant compared with the control value (*P*<0.05).

It is known that the effect of Ang II could be lost due to impairment of the coupled signaling pathways [14]. Therefore, in a further group of experiments we tested the chronic effect of Ang II by treating the culture daily with 10^−8^ M Ang II for 4 consecutive days (2 complete parasite life cycles) with an initial inoculum of 0.5%. The inhibitory effect of Ang II was maintained throughout the experiment (Fig. 2A). One question arises from our data: is the effect of Ang II on parasite growth maintained even in the absence of a new dose of Ang II? In another group of experiments, infected erythrocytes (0.5% parasitemia) were pre-incubated or not with daily doses of Ang II (10^−8^ M) for 4 consecutive days, as described before. The culture was then split and maintained for 11 days without Ang II. Parasitemia was then evaluated. The pre-incubation treatment decreased parasitemia compared with the control (no pre-incubation with Ang II, Fig. 2B, bar A), represented by 50% inhibition (Fig. 2B, bar B). The inhibitory effect of Ang II is maintained even after 11 days in the absence of a new addition of Ang II (Fig. 2B, bar C).

### Erythrocytes express angiotensin receptors

**Figure 3:**
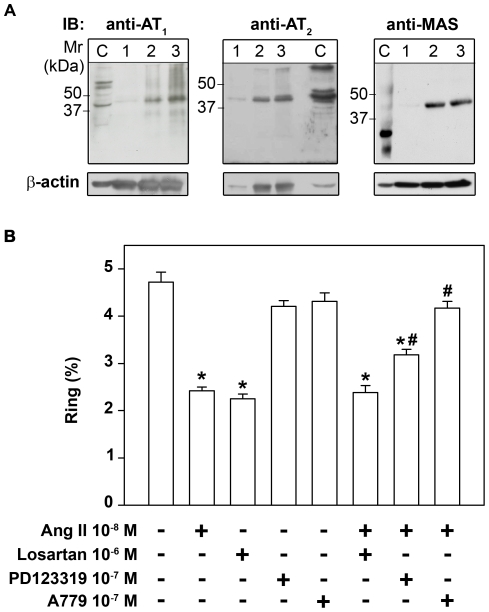
Angiotensin receptors are present in erythrocyte membranes. (A) Membrane preparations of human erythrocytes (50, 150 or 200 µg total protein, lanes 1, 2 and 3, respectively) underwent immunoblotting for detection of AT_1_, AT_2_ and MAS receptors. The microsomal fraction of rat renal cortex was used as positive control (lane C) (n = 3). (B) The Ang II-mediated effect was partially abolished by AT_2_ receptor antagonist. Where indicated, cultures were pre-treated with 10^−6^ M losartan, 10^−7^ M PD123319 or 10^−7^ M A779. Parasite invasion was determined as the number of intracellular rings after 24 h incubation as described in the Materials and Methods section (n = 6). The results are expressed as means±SE. Statistically significant compared with *control and #Ang II values (*P*<0.05).

To verify the presence of Ang II receptors on the surface of erythrocytes, membrane fractions were subjected to immunoblotting analysis. Rat renal cortex homogenate was used as positive control because it is well known that this preparation has AT_1_, AT_2_ and MAS angiotensin receptors [15]–[17]. Fig. 3A clearly shows the presence of the same receptors in erythrocyte plasma membranes. The molecular weight found for AT_1_ was a little higher than that observed in Wistar rat renal cortex homogenate (43 kDa), the positive control used. In agreement with our results, AT_1_ receptor isoforms with molecular weight ranging from 60 to 73 kDa have been observed in the kidneys of the Sprague-Dawley rat, desert rodent *Meriones crassus* and human ovarian carcinoma cells [18], [19].

To investigate which receptor was mediating the Ang II effect, cells were treated with 10^−6^ M losartan, 10^−7^ M PD123319 or 10^−7^ M A779, antagonists of AT_1_, AT_2_ and MAS receptors, respectively. These antagonists were added 30 min before the addition of Ang II and maintained during the course of the reaction with Ang II. The inhibitory effect of Ang II on parasite invasion was partially abolished when PD123319 was added and completely abolished in the presence of A779. The addition of PD123319 or A779 without Ang II did not change the parasite invasion (Fig. 3B). On the other hand, losartan had the same but non-additive effect compared with Ang II.

### Ang II-derived metabolites mimicked the effect of Ang II on parasite invasion

**Figure 4:**
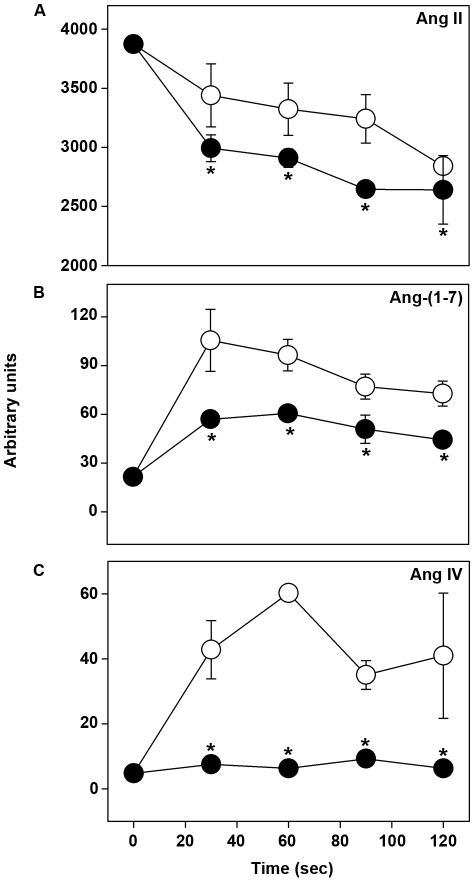
Ang II is promptly metabolized during the invasion assay. The time course of Ang II metabolism was monitored by mass spectroscopy (MALDI) in the supernatant of infected (•) and non-infected (○) erythrocytes incubated with 10^−5^ M Ang II. (A) Ang II, (B) Ang-(1–7) and (C) Ang IV levels. The peak areas for different angiotensin forms (Ang II, Ang-(1–7) and Ang IV) present in the same spectrum (masses 1046.19, 899.02 and 774.92 Da, respectively) were obtained and their sum was arbitrarily assigned as 100%. The results are expressed as means±SE. *Statistically significant compared with the control value (n = 4, *P*<0.05).

**Figure 5:**
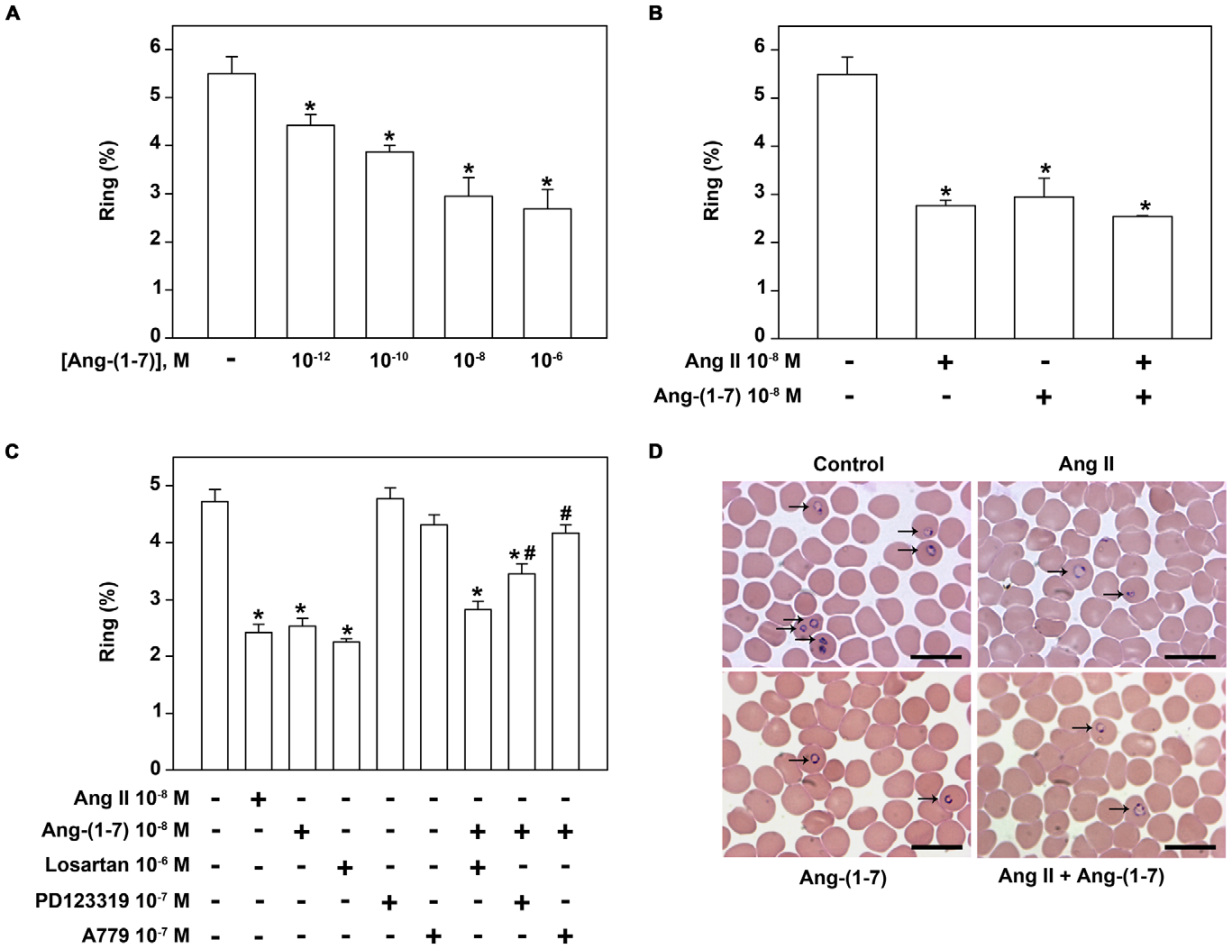
Ang-(1–7) has the same but non-additive effect as Ang II. (A) Dose-response of the effect of Ang-(1–7) on the *P. falciparum* erythrocytic cycle. Parasite schizont forms were incubated with a fresh erythrocyte culture at 2% parasitemia in the absence or presence of increasing concentrations of Ang-(1–7) (10^−12^–10^−6^ M). Parasite invasion was determined as the number of intracellular rings after 24 h incubation as described in the Materials and Methods section (n = 8). (B) Parasite schizont forms were incubated with a fresh erythrocyte culture at 2% parasitemia in the absence or presence of 10^−8^ M Ang II, 10^−8^ M Ang-(1–7) or both. Parasite invasion was determined as the number of intracellular rings after 24 h incubation as described in the Materials and Methods section (n = 8). (C) The Ang-(1–7)-mediated effect was avoided by the MAS receptor antagonist. The effect of Ang II in parasite invasion was determined as described in (B). Where indicated, cultures were pre-treated with 10^−6^ M losartan, 10^−7^ M PD123319 or 10^−7^ M A779 before addition of Ang-(1–7) (10^−8^ M). Parasite invasion was determined as the number of intracellular rings after 24 h incubation as described in the Materials and Methods section (n = 6). (D) The effect of Ang II and Ang-(1–7) on the erythrocytic cycle of malaria parasite obtained from thick blood smears for parasitemia determination by Diff-Quick staining (n = 8). The results are expressed as means±SE. Statistically significant compared with *control and #Ang-(1–7) values (*P*<0.05). Magnification ×100.

So far the scenario indicates that Ang II could modulate parasite invasion. However, it is well known that Ang II can be metabolized and, consequently, the metabolites generated could be active in the modulation of the *P. falciparum* erythrocytic cycle. To address this issue, we measured Ang II metabolism using mass spectroscopy (Fig. 4). Initially, infected and non-infected erythrocytes were incubated with 10^−5^ M Ang II for different times (30–120 s). The supernatant was collected and analyzed by mass spectroscopy as described in the Materials and Methods section. We assessed the level of aminopeptidase-mediated Ang IV and carboxypeptidase-mediated Ang-(1–7) formation because these peptides have been reported to have important physiological effects. The basal levels of Ang II, Ang IV and Ang-(1–7) in the serum were less than 1% compared with the levels found after the addition of 10^−5^ M Ang II. Ang II is metabolized under both conditions (Fig. 4A). However, its metabolism is significantly lower in infected erythrocytes than in non-infected ones. In non-infected erythrocytes, aminopeptidase-mediated Ang IV and carboxypeptidase-mediated Ang-(1–7) formation occurred. However, in infected erythrocytes, a decrease in the level of Ang-(1–7) and complete inhibition of the formation of Ang IV were observed, indicating that the metabolic pathways are under differential regulation in the presence of the parasite (Fig. 4B,C). Thus, these results suggest that the effect of Ang II on the erythrocytic parasite cycle could be mediated by its carboxypeptidase-mediated metabolic product Ang-(1–7). We then tested whether or not the effect of Ang II could be due to the formation of carboxypeptidase-mediated Ang-(1–7). As observed for Ang II, when increasing concentrations of Ang-(1–7) (10^−12^–10^−6^ M) were added to the invasion assay, parasite invasion was reduced in a dose-dependent manner with the maximum effect observed at a concentration of 10^−8^ M, achieving 41% of inhibition (Fig. 5A) 24 h after treatment. Ang-(1–7) concentrations as low as 10^−12^ and 10^−10^ M had a significant inhibitory effect (*P*<0.005 and *P*<0.0001, respectively). When both Ang II and Ang-(1–7) were assayed concomitantly against the *P. falciparum* culture, the same level of inhibition was observed, demonstrating a non-additive effect of both peptides (Fig. 5B,D). A779 (10^−7^ M), a specific antagonist of MAS receptor, known to transduce Ang-(1–7) signaling, completely reversed the effect of Ang-(1–7). PD123319 (10^−7^ M) was also able to partially reduce the inhibitory effect of Ang-(1–7) on the parasite infection. On the other hand, losartan (10^−6^ M) did not change the inhibitory effect of Ang-(1–7) (Fig. 5C). Thus, the effects of angiotensin receptor antagonists on the inhibitory effect of Ang-(1–7) were similar to those observed in the presence of Ang II (Figs. 3B and 5C).

**Figure 6:**
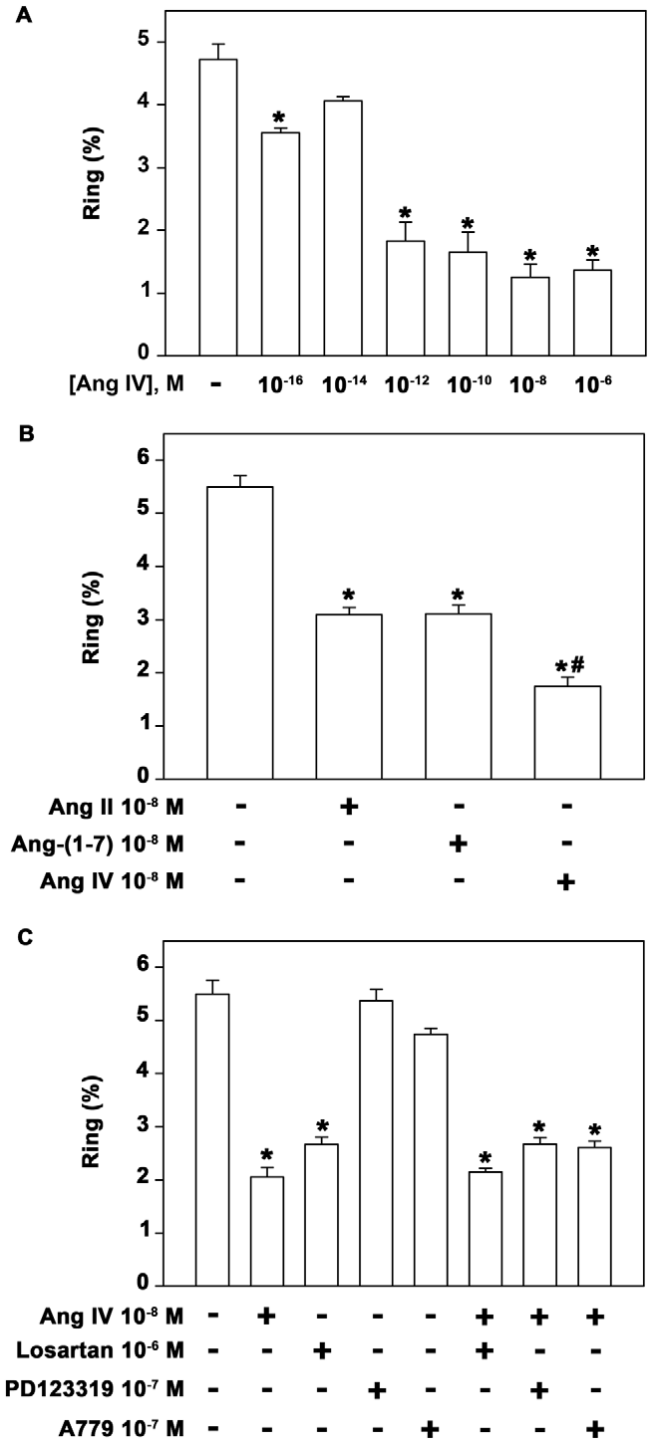
Ang IV has a greater effect than Ang II and Ang-(1–7). (A) Dose–response of the effect of Ang IV on the *P. falciparum* erythrocytic cycle. Parasite schizont forms were incubated with a fresh erythrocyte culture at 2% parasitemia in the absence or presence of increasing concentrations of Ang IV (10^−12^–10^−6^ M). Parasite invasion was determined as the number of intracellular rings after 24 h incubation as described in the Materials and Methods section (n = 5). (B) Parasite schizont forms were incubated with a fresh erythrocyte culture at 2% parasitemia in the absence or presence of 10^−8^ M Ang II, 10^−8^ M Ang-(1–7) or 10^−8^ M Ang IV. Parasite invasion was determined as the number of intracellular rings after 24 h incubation as described in the Materials and Methods section (n = 5). (C) The Ang IV-mediated effect was not prevented by AT_1_, AT_2_ or MAS receptor antagonists. The effect of Ang IV on parasite invasion was determined as described in (A). Where indicated, cultures were pre-treated with 10^−6^ M losartan, 10^−7^ M PD123319 or 10^−7^ M A779 before addition of Ang IV (10^−8^ M). Parasite invasion was determined as the number of intracellular rings after 24 h incubation in thick blood smears as described in the Materials and Methods section (n = 5). The results are expressed as means±SE. Statistically significant compared with *control value and #Ang II and Ang-(1–7) (*P*<0.05).

Although Ang IV formation is inhibited in infected erythrocytes, we decided to verify the influence of Ang IV on the parasite erythrocytic cycle (Fig. 6) because the results could provide important clues as to why the parasite induces the inhibition of Ang IV formation. The dose–response relationship between Ang IV and the parasite erythrocytic cycle showed that the peptide inhibited infection of new erythrocytes by 70% with the maximum effect observed at lower doses (10^−12^ M) (Fig. 6A). Ang II and Ang-(1–7) achieved the maximum inhibitory effect only at 10^−8^ M. Ang IV has a more potent inhibitory effect compared with Ang-(1–7) and Ang II (Fig. 6B) and its effect was not modified by any of the angiotensin receptor antagonists used: 10^−7^ M PD123319, 10^−7^ M A779 and 10^−6^ M losartan (Fig. 6C).

### Ang-(1–7)-induced inhibition involves MAS-mediated reduction of erythrocyte PKA activity

**Figure 7:**
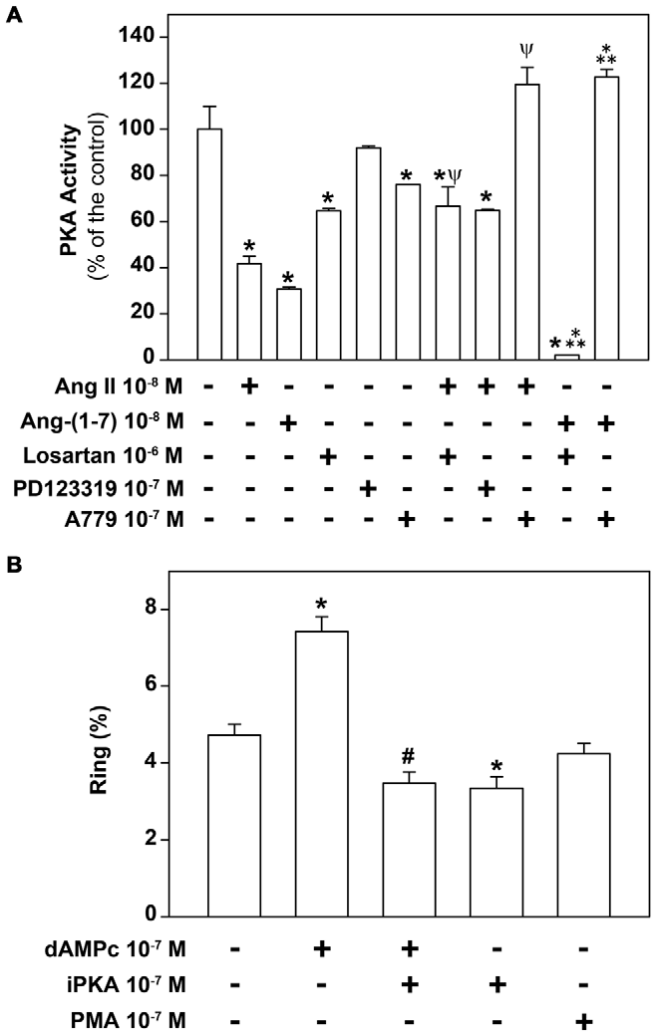
Erythrocyte PKA is reduced by Ang II and Ang-(1–7) and is involved in infection. (A) The membrane fraction of erythrocytes was assayed for PKA activity as described in the Materials and Methods section, in the absence or presence of 10^−8^ M Ang II or 10^−8^ M Ang-(1–7). Where indicated, membranes were pre-treated with 10^−6^ M losartan, 10^−7^ M PD123319 or 10^−7^ M A779 before the addition of 10^−8^ M Ang II or 10^−8^ M Ang-(1–7) (n = 6). (B) Parasite schizont forms were incubated with a fresh erythrocyte culture at 2% parasitemia in the absence or presence of 10^−7^ M dAMPc, 10^−7^ M iPKA or 10^−7^ M PMA. Parasite invasion was determined as the number of intracellular rings after 24 h incubation as described in the Materials and Methods section (n = 7). The results are expressed as means±SE. Statistically significant compared with *control value, ^ψ^Ang II, ^Ψ^Ang-(1–7), ^#^dAMPc (*P*<0.05).

It is known that erythrocyte Gs protein is a crucial player during the malaria blood cycle [8]. Furthermore, it has been shown that Ang II and Ang-(1–7) can modulate PKA activity in different cell types [17]. It is well known that erythrocytes display different kinase activities, including PKA [20]. Thus, we decided to investigate if erythrocyte PKA could be involved in the effect of these peptides on the parasite erythrocytic cycle. Because *P. falciparun* also expresses PKA (PfPKA) we measured the effect of Ang II or Ang-(1–7) on erythrocyte PKA activity using the membrane preparation (Fig. 7). In this way, any influence of PfPKA is diminished and the investigation is directed to possible changes in erythrocyte activity. We found that 10^−8^ M Ang II or 10^−8^ M Ang-(1–7) inhibited the basal PKA activity and this effect was reversed by 10^−7^ M A779 (Fig. 7A). In addition, by evaluating the number of parasite ring forms, we showed that 10^−6^ M cAMP permeable analogue (dibutyryl-cAMP) increased the level of infection (Fig. 7B). On the other hand, 10^−7^ M PKA inhibitor not only reversed the stimulation induced by dibutyryl-AMPc but also significantly decreased the parasite erythrocytic cycle by 30% when added alone in the interaction assay (Fig. 7B). 10^−12^ M phorbol myristate acetate (PMA), a well-known protein kinase C (PKC) activator, did not change the level of infection.

## Discussion

The invasive stages of the malaria parasite are considered attractive targets for the development of antimalarial drugs and vaccines, especially the merozoite invasion of erythrocytes during the *P. falciparum* blood stage [9]. Although different invasion pathways between erythrocytes and *Plasmodium* sp. have been well described, the physiological aspects involving host components in this process are still poorly understood. Our results provide the first evidence of the important role of RAS components in erythrocyte infection by *P. falciparum*. Here we show that Ang II, through its carboxypeptidase-mediated metabolic product, Ang-(1–7), decreases infection of new erythrocytes during development of the *P. falciparum* blood stage. This effect of Ang-(1–7) is specific and probably involves a MAS-mediated PKA inhibition.

The first question that arises from our results is: What is the source of Ang II involved in the modulation of the erythrocyte cycle of *P. falciparum*? Recently, local tissue generation of Ang II has been described; this peptide could have paracrine or autocrine effects [21]–[24]. Therefore, it is possible to postulate that Ang II produced locally by different tissues such as immune and vascular cells could modulate the erythrocyte cycle of *P. falciparum*.

The observation that the effects of Ang II on *P. falciparum* blood stage are mediated by Ang-(1–7) reveals a new role for the (ACE2)/Ang-(1–7)/MAS receptor axis. This idea is strengthened by the observation that concentrations of Ang-(1–7) that inhibit parasite invasion (10^−12^–10^−10^ M) are compatible with the physiological serum concentration of Ang-(1–7) [25], [26].

It is well known that Ang II promotes endothelial dysfunction [27], which could be associated with malaria pathogenesis. In this context, in a study on patients in Orissa/India, Dhangadamajhi et al. [12] showed that the D allele of ACE I/D polymorphism and ACE2 C→T substitution, responsible for high level of Ang II in serum, are associated with mild malaria. The authors postulated that the protective effect of Ang II could be explained by its antiplasmodial activity based on the observations of Maciel et al. [11] in *Aedes aegypti* infected with *Plasmodium gallinaceum*. However, the mechanism involved in antiplasmodial activity in the human host has not yet been determined. In the present study, we showed that the antiplasmodial effect of Ang II on the *P. falciparum* erythrocytic cycle is mediated by its conversion into Ang-(1–7).

An important question regarding the effect of angiotensin peptides on the *P. falciparum* erythrocytic cycle is what is the molecular mechanism involved in this process? The possibility of explaining our results based on the effect of Ang II on the plasma membrane structure, as shown by Maciel et al. [11], seems to be ruled out. The effects of Ang II and Ang-(1–7) on the *P. falciparum* erythrocytic cycle were completely abolished by specific receptor antagonists, indicating a correlation between the structure and function of angiotensin peptides in the effect observed in the present work.

Here, we clearly show the presence of angiotensin receptors, AT_1_, AT_2_ and MAS (or AT_(1–7)_), in erythrocyte membranes, and their selective blockage by specific antagonists revealed important clues about their involvement in parasite invasion. The observation that Ang II and Ang-(1–7) effects are blocked by A779 and not changed by losartan show the involvement of the MAS receptor in the modulation of the *P. falciparum* erythrocytic cycle. This agrees with the observation that Ang II is metabolized to Ang-(1–7) in non-infected or even in infected cultures in which Ang-(1–7) formation is reduced. In addition, the effect of these peptides was partially abolished by the AT_2_ receptor antagonist, PD123319. These results indicate that MAS and AT_2_ receptors could be working in a coordinated manner to mediate the effect of Ang-(1–7). Similar results have been obtained in other systems such as in the modulation of blood pressure where the effect of Ang-(1–7) depends on the interaction of both receptors [28], [29]. The lack of response of losartan, PD123319 and A779 on Ang IV-induced inhibition of parasite invasion demonstrates that the effects of this peptide could be mediated by a specific receptor for Ang IV (IRAP or AT_4_) [23].

Angiotensin peptide receptors AT_1_, AT_2_ and MAS belong to the GPCR family. MAS receptor was recently cloned, but the signaling pathways activated by this receptor are not well established [30]. Here we observed that Ang II and Ang-(1–7) inhibit PKA activity in an A779-sensitive manner similar to inhibition of the *P. falciparum* erythrocytic cycle. In agreement with the role of PKA in the *P. falciparum* erythrocytic cycle, it was observed that PKA inhibitor was also able to inhibit the infection, whereas the permeable analogue of cAMP increases it. This effect is specific for PKA because the activation of PKC by PMA did not change the *P. falciparum* erythrocytic cycle. These results indicate that the inhibitory effect of Ang-(1–7) on the *P. falciparum* erythrocytic cycle is mediated by the inhibition of PKA. *P. falciparum* PKA (PfPKA) has been recognized to have an important role in the development of blood-stage parasites with highest activity in schizonts [10]. PfPKA modulates anion channel conductance in erythrocytes, which is important for parasite growth. However, we measured PKA activity in non-infected erythrocytes and showed that Ang-(1–7) inhibits the *P. falciparum* erythrocytic cycle by modulating erythrocyte PKA activity. In addition, the α subunit of the Gs protein (Gαs) was found in detergent-resistant rafts of erythrocyte membrane [7]. The Gs protein-associated signaling mechanisms triggered by two different GPCR agonists stimulated malaria infection and parasite intracellular maturation in agreement with our results [8].

Together, the results obtained here reveal a potential role of RAS in the *P. falciparum* blood stage. These results open new avenues for the development of new antimalarial drugs.

## Materials and Methods

### Ethics statement

Collection of human blood samples for this study was conducted according to the protocols approved by the Research Ethics Committee of the Hospital Universitário Clementino Fraga Filho from Federal University of Rio de Janeiro (Permit Number 074/10). All patients provided written informed consent for the collection of samples and subsequent use. The use of this material follows long-standing protocols and has not been associated with any adverse or other unforeseen events and no data of relevance to specific patients has been generated. Also, this study was carried out in strict accordance with the recommendations in the Guide for the Care and Use of Laboratory Animals of the National Institutes of Health. The protocol was approved by the Institutional Ethics Committee of Federal University of Rio de Janeiro (Permit Number IBCCF098).

### Compounds

*N*-2-Hydroxyethylpiperazine *N*-2-ethanesulfonic acid (HEPES), (tris-hydroxymethyl)-aminomethane (Tris), glucose, sodium bicarbonate, hypoxanthine, adenosine triphosphate (ATP, sodium salt), magnesium chloride, ethylenediaminetetraacetic acid (EDTA), histone, protein kinase A inhibitor peptide (PKAi), PD123319, Ang II (Asp-Arg-Val-Tyr-Ile-His-Pro-Phe), Ang IV (Val-Tyr-Ile-His-Pro-Phe) and Ang-(1–7) (Asp-Arg-Val-Tyr-Ile-His-Pro) were purchased from Sigma Aldrich Co. (St. Louis, MO, USA). Percoll was purchased from GE Healthcare (Uppsala, Sweden). [^32^Pi]Pi was obtained from the Brazilian Institute of Energetic and Nuclear Research, São Paulo, Brazil. [γ-^32^Pi]ATP was synthesized according to the procedures described by Maia et al. [31]. The AT_1_ receptor selective antagonist, losartan, was obtained from Medley S.A. (São Paulo, Brazil) and A779, the MAS receptor blocker, was kindly provided by Dr Robson Augusto Souza dos Santos (Department of Physiology and Biophysics, Institute of Biological Sciences, Federal University of Minas Gerais, MG, Brazil). All other reagents were of the highest purity available.

### Parasite culture in vitro

Erythrocytic asexual stages of *P. falciparum* W2 strain, characterized as chloroquine resistant and mefloquine sensitive [32], were maintained in continuous culture in RPMI 1640 medium (Invitrogen, CA, USA) in the presence of A-type human blood and 10% A-positive human serum, 50 µg/ml gentamicin (Invitrogen, CA, USA) at 4–5% hematocrit, as described by Trager and Jensen [33]. A thick blood smear was prepared for parasitemia determination by Diff-Quick staining and % parasitemia was described as the number of parasitized red blood cells (PRBC) in 100 erythrocytes, after analysis of at least five random microscopic fields.

### Parasite invasion assay

Parasite mature forms were collected using a Percoll/sorbitol gradient as described in standard protocols [34]. Briefly, a suspension of PRBC at 20% hematocrit was centrifuged at 3600 rpm for 30 min at room temperature in a bench top centrifuge over a 40%, 70% and 90% Percoll/sorbitol gradient. The brown band containing more than 90% PRBC formed at the 40% and 70% interface was harvested and used in interaction assays with fresh erythrocyte cultures at indicated parasitemia levels in the presence or absence of specific compounds as described in the figure legends. Alternatively, cultures enriched in the shizont form were obtained after synchronization with 5% sorbitol as described elsewhere [35].

### Mass spectrometry analysis (MALDI)

C18 ZipTip micropipette tips were used for desalting angiotensin peptides in the medium. The tips were first activated with acetonitrile and equilibrated with 0.1% (v/v) trifluoroacetic acid (TFA) in water. The samples were aspirated and dispensed for eight cycles and the tips were washed with 0.1% (v/v) TFA in water three times. The peptides retained on the tips were eluted using 1.5 µl of a mixture of 50% acetonitrile and 0.1% (v/v) TFA in water. Then, 0.3 µl of this eluate was immediately spotted on the ABI 192-target MALDI plate (Applied Biosystems, USA) by cocrystallization with 0.3 µl of an α-cyano-4-hydroxycinnamic acid matrix (10 mg/ml in 30% acetonitrile, 0.3 (v/v) TFA in water). Raw data for angiotensin forms were obtained on the 4700 Proteomics Analyzer (Applied Biosystems, Foster City, CA). Mass spectrometry data were acquired in positive and reflectron mode, mass range 600–1100 Da, using a neodymium-doped yttrium aluminum garnet (Nd:YAG) laser with a 200-Hz repetition rate. Typically, 1600 shots were accumulated for spectra. External calibration was performed using a mixture of four peptides: des-Arg1-bradykinin (*m*/*z* = 904.47), angiotensin I (*m*/*z* = 1296.69), Glu1-fibrinopeptide B (*m*/*z* = 1570.68), and adrenocorticotropic hormone (18–39) (*m*/*z* = 2465.20). The spectrum files (T2D) were generated from the raw (or native) mass spectrometry data using Data Explorer Software v.4.8 (Applied Biosystems). To monitor angiotensin metabolism, the areas of the peaks for different angiotensin forms (Ang II, IV and 1–7) present in the same spectrum, masses 1046.19, 774.92 and 899.02 Da, respectively, were obtained and their summation was arbitrarily assigned as 100%, making it possible to observe the relative changes of each form as a function of time.

### Erythrocyte membrane preparation and immunodetection of angiotensin receptors

Erythrocyte membranes were prepared from fresh human A^+^ blood, obtained from consenting adult donors, lysed isotonically by freeze–thaw cycle in liquid nitrogen as described previously [36]. Briefly, blood was filtered in gauze before centrifugation in a Sorvall centrifuge at 6871×*g*, for 10 min, at 4°C. The pellet was re-suspended in 20 mM Tris–130 mM KCl buffer (pH 7.4), and cells were lysed by freezing in liquid nitrogen and thawing naturally at room temperature. The resulting fluffy pellet was re-suspended and homogenized in 5 mM Hepes–1 mM EDTA buffer (pH 7.4), and hemoglobin was washed out by centrifugation at 10,409×*g*, for 17 min, at 4°C. A second wash was carried out using 5 mM Hepes buffer (pH 7.4) and then a final wash with a buffer containing 10 mM Tris, 130 mM KCl, 0.5 mM MgCl_2_ and 50 mM CaCl_2_. The clarified preparation was re-suspended in a minimal amount of the same buffer. The final protein concentration in this enriched membrane fraction was determined by the Folin phenol method [37] using bovine serum albumin as a standard.

The membrane preparation was re-suspended and lysed on ice for 20 min in Ripa buffer (25 mM Tris–HCl, pH 7.5, 150 mM NaCl, 1 mM EDTA, 1% Triton X-100, 0.5% deoxycholate and 0.1% sodium dodecyl sulfate) freshly supplemented with phosphatase and protease inhibitors (10 mM NaF, 5 mM Na_3_VO_4_, 5 mM Na_4_P_2_O_7_ and 1× protease inhibitor cocktail, Roche, IN, USA). Aliquots containing 50, 150 or 200 µg protein were re-suspended in SDS-PAGE loading buffer, resolved on SDS 9% acrylamide gels and transferred onto Immobilon-P membranes (Millipore, MA, USA). After blocking with 5% non-fat dry milk/Tris-buffered saline containing 0.1% Tween 20 for 1 h at room temperature, membranes were probed overnight, at 4°C, with primary specific antibodies, followed by horseradish peroxidase-labeled secondary antibodies (Amersham Biosciences) and visualized with ECL®-plus reagent (Enhanced Chemiluminescence, Amersham Biosciences). Rabbit polyclonal anti-human MAS1 was purchased from Abcam (MA, USA); mouse monoclonal anti-human AT_1_ and rabbit polyclonal anti-human AT_2_ were obtained from Santa Cruz Biotechnology, Inc. (CA, USA).

### Measurement of PKA activity

The PKA activity of isolated erythrocyte membranes was measured by protein kinase inhibitor-sensitive incorporation of [^32^Pi]Pi from [γ-^32^P]ATP (7 µCi/µmol), using histone as substrate. The composition of the reaction medium was 4 mM MgCl_2_, 20 mM HEPES–Tris (pH 7.0), 1.5 mg/ml histone and 0.7 mg/ml protein. After 10 min, the reaction was stopped with 40% trichloroacetic acid (TCA) and the sample was immediately placed on ice. An aliquot (0.1 ml) was filtered through a Millipore filter (0.45 µm pore size) and washed with ice-cold 20% TCA solution and 0.1 M phosphate buffer (pH 7.0). The radioactivity was quantified using a liquid scintillation counter. The specific PKA activity was calculated from the difference between the activity in the absence and in the presence of 10^−8^ M PKAi [38].

### Statistical analysis

Each experiment was performed in independent cell suspensions. The data were analyzed by two-way analysis of variance (ANOVA), considering the treatments as factors. The significance of the differences was verified by the Bonferroni *t*-test. Statistical analysis was performed using absolute values.
